# Durability of switch regimens based on rilpivirine or on integrase inhibitors, both in association with tenofovir and emtricitabine, in HIV-infected, virologically suppressed patients

**DOI:** 10.1186/s12879-017-2831-9

**Published:** 2017-11-16

**Authors:** Nicola Gianotti, Andrea Poli, Silvia Nozza, Laura Galli, Nadia Galizzi, Marco Ripa, Marco Merli, Alessia Carbone, Vincenzo Spagnuolo, Adriano Lazzarin, Antonella Castagna

**Affiliations:** 1 Infectious Diseases, San Raffaele Scientific Institute, Via Stamira d’Ancona 20, 20127 Milan, Italy; 2 Università Vita-Salute San Raffaele, Milan, Italy

**Keywords:** Rilpivirine, Raltegravir, Elvitegravir/cobicistat, Dolutegravir, Non-nucleoside reverse transcriptase inhibitors, Integrase inhibitors, Switch regimen, Residual viremia, Virological suppression

## Abstract

**Background:**

Switch strategies based on rilpivirine/tenofovir/emtricitabine or on an integrase inhibitor (InSTI) plus tenofovir/emtricitabine have never been compared in randomized clinical trials. The main aim of the study was to investigate the durability of these two switch regimens in virologically suppressed, HIV-infected patients.

**Methods:**

Retrospective analysis of patients who started rilpivirine or an InSTI (both with tenofovir and emtricitabine) with <50 HIV-RNA copies/mL and had at least one HIV-RNA assessed while receiving the study regimen. Virological failure (VF) was defined as two consecutive measurements of HIV-RNA >50 copies/mL. Treatment failure (TF) was define as either VF or discontinuation of any drug of the regimen. Durability was assessed by the Kaplan-Meier method and compared by Log-rank test. Residual viremia was defined as any detectable HIV-RNA below 50 copies/mL, as assed by a real-time PCR assay.

**Results:**

Six hundred seventy-five patients (466 switched to a rilpivirine-, 209 switched to an InSTI-based regimen [18% dolutegravir, 39% raltegravir, 43% elvitegravir/cobicistat] were included in the analysis.

The median (interquartile range, IQR) follow-up in the rilpivirine and in the InSTI group was 16.7 (8.8–22.2) and 10.4 (5.4–19.6) months. The 1-year cumulative probabilities (95%CI) of VF and TF were 0.97% (0.36%–2.62%) and 9.73% (7.21%–13.06%) in the rilpivirine group and 1.83% (0.57%–5.77%) and 8.75% (5.25%–14.4%) in the InSTI group, with no difference between groups (*p* = 0.328 and 0.209 for VF and TF). The proportion of time spent with residual viremia was comparable in the two groups (9% [IQR 0.5%–49%] and 17% [IQR 0.5%–50%] in the rilpivirine and in the InSTI group, *p* = 0.087).

By the multivariable Cox regression model, TF was independently associated with being on therapy with a protease inhibitor vs. a non-nucleoside reverse transcriptase inhibitor at switch (AHR = 0.52; 95%CI = 0.31–0.90; *p* = 0.018), baseline total/HDL-cholesterol ratio (AHR = 1.19 per 0.5-units increments; 95%CI = 1.06–1.34; *p* = 0.004), baseline estimated glomerular filtration rate (AHR = 0.78 per 10-units increments; 95%CI = 0.67–0.90; *p* = 0.001) and baseline hemoglobin (AHR = 0.78 per 1-unit increments; 95%CI = 0.64–0.94; *p* = 0.009), but not with treatment group (rilpivirine vs. InSTI).

**Conclusions:**

In our clinical practice, the durability of the two regimens was comparable and both showed a very low probability of VF.

**Electronic supplementary material:**

The online version of this article (10.1186/s12879-017-2831-9) contains supplementary material, which is available to authorized users.

## Background

Randomized clinical trials support the switch both to the fixed dose combination (FDC) of rilpivirine/tenofovir disoproxil fumarate/emtricitabine from a ritonavir-boosted protease inhibitor (PI/r) and to regimens based on integrase strand transfer inhibitors (InSTI) from any kind of antiretroviral therapy. In the SPIRIT Study switching from a regimen based on a PI/r to the FDC of rilpivirine/tenofovir/emtricitabine was not inferior to continuing the PI/r, with virological success in 93,7% and 89,9% of patients at 48 weeks [1]. Non-inferiority was shown also for switching from the FDC of efavirenz/tenofovir disoproxil fumarate/emtricitabine to rilpivirine/tenofovir alafenamide/emtricitabine [2], as well as for switching from a PI/r to raltegravir [3], from a regimen based on a non-nucleoside reverse transcriptase inhibitor (NNRTI) to the FDC of elvitegravir/cobicistat/emtricitabine/tenofovir disoproxil fumarate [4] or from any regimen to the FDC of dolutegravir/abacavir/lamivudine [5]. By contrast, in the STRATEGY-PI Study, switching from a PI/r to the FDC of elvitegravir/cobicistat/emtricitabine/tenofovir disoproxil fumarate was superior in terms of virological success (93,8% vs. 87,1%) and improved patients related outcomes [6], while switching from lopinavir/ritonavir to raltegravir (without changing the nucleoside backbone) was associated with a higher incidence of virological failure [7].

However, these switch strategies have never been compared in randomized clinical trials and it is very unlikely that such a trial will be performed.

The aim of this study was two-fold: first, we aimed at identifying factors associated with the choice of a FDC of rilpivirine/tenofovir/emtricitabine vs. an InSTI-based switch regimen; secondly, we aimed at investigating the durability of rilpivirine/tenofovir/emtricitabine and of InSTI-based switch regimens (when associated to tenofovir disoproxil fumarate/emtricitabine, TDF/FTC) in virologically suppressed HIV-infected patients in clinical practice.

## Methods

Retrospective cohort study on patients followed at the Infectious Diseases Department of the San Raffaele Scientific Hospital in Milan (Italy). Data recorded in the database of the Infectious Diseases Department of the San Raffaele Hospital (IDD-HSR) were used for the analyses. At their first visit in our clinic, subjects provide written informed consent to include their clinical and laboratory data in the IDD-HSR for scientific purposes. The study was approved by the ethics committee of the San Raffaele Scientific Institute.

Eligible patients were those who started rilpivirine or an InSTI (both along with TDF and FTC) with <50 HIV-RNA copies/mL and had at least one HIV-RNA assessed while receiving the study regimen. Patients who switched to one of the study regimens since 2008 (date of availability of raltegravir in Italy) were included in the analyses. Patients were followed up to virological failure (VF) or discontinuation of any drug or data freezing (8th September 2015), whichever occurred first.

Estimated glomerular filtration rate (eGFR) was calculated using the Chronic Kidney Disease Epidemiology Collaboration Equation (CKD-EPI) [8] and the liver fibrosis FIB-4 index was calculated as described [9].

HIV-RNA was quantified by using the kinetic PCR molecular system (kPCR, Versant HIV-1 RNA kPCR 1.0; Siemens HealthCare Diagnostics, Tarrytown, NY, USA) up to March 2014 and by using the Abbot Real-Time PCR (Abbott Molecular, Des Plaines, IL, USA) thereafter. The kPCR assay gives three possible outputs: (i) a quantitative result for HIV-RNA values of ≥37 copies/mL; (ii) a semiquantitative result (detectable below 37 copies/mL) when HIV RNA is detectable but not precisely quantifiable; (iii) a qualitative result (‘undetectable’) when no signal can be detected. The Abbot Real-Time PCR assay gives also three possible outputs: (i) a quantitative result for HIV RNA values of ≥40 copies/mL; (ii) a semiquantitative result (detectable below 40 copies/mL) when HIV RNA is detectable but not precisely quantifiable; (iii) a qualitative result (‘undetectable’) when no signal can be detected.

VF was defined as two consecutive measurements of HIV-RNA >50 copies/mL or a single HIV-RNA >50 copies/mL followed by ART modification; an unconfirmed HIV-RNA >50 copies/mL (i.e. one measurement >50 copies/mL preceded and followed by a measurement <50 copies/mL), not followed by ART modification, was defined as viral blip. Residual viremia was defined as any detectable HIV-RNA below 50 copies/mL, as assed by Siemens kPCR or by Abbot Real-Time PCR.

Time spent with residual viremia was calculated as a proportion of time with residual viremia on observed follow-up. If between 2 observation the viremia changed from undetectable to residual or vice-versa, the time spent considered in this interval was the half. The mathematical formula was:

$$ {T}_{\%}=\frac{\sum_{i=1}^{i=j}\left(\frac{t_i-{t}_{i-1}}{a}\right)}{t_{tot}}\cdot 100 $$

where *t* is the time length of interval *i, j* is the last observation and *t*_*tot*_ is the patient’s cumulative follow-up. If, during the *i* ^*th*^ interval, viremia changed from undetectable to residual or vice-versa*,* then *a = 2,* else *a* = 1.

Treatment failure (TF) was define as either VF or discontinuation (for any length) of any drug of the regimen, for any reason. Causes of change in the regimen were reviewed independently by two clinicians; discordances were discussed and reconciled.

Descriptive data are expressed as median (interquatile range) of frequency (%), as appropriate.

Chi-square and Mann-Whitney tests were used to evaluate differences between the two groups for categorical and continuous variables respectively. Durability was assessed by the Kaplan-Meier curve and compared by Log-rank test. Multivariable logistic regression was used to identify predictors of opting for an InSTI- rather than a rilpivirine-based regimen and a multivariable Cox regression model was used to identify factors independently associated with TF.

All of the statistical tests were two-sided at 5% level, and were performed using SAS Software (release 9.2; SAS Institute).

## Results

Six hundred seventy-five patients (466 switched to a rilpivirine-, 209 switched to an InSTI-based regimen [18% dolutegravir, 39% raltegravir, 43% elvitegravir/cobicistat]), on antiretroviral therapy since 6.6 [3.3–14.1] years and with HIV-RNA <50 copies/mL since 3.1 [1.1–5.6] years, were included in the analysis; their baseline characteristics are illustrated in Table 1 and in the Additional file 1: Table S1. Dolutegravir was used at a daily dose 50 mg in all cases, raltegravir at the standard dose of 400 mg twice daily and elvitegravir/cobicistat was always co-formulated with TDF/FTC).

**Table 1:** Baseline characteristics

	Overall (*n* = 675)	InSTI + TDF/FTC (*n* = 209)	RPV/FTC/TDF (*n* = 466)	*p*-value
Age *(years)*	46.2 (39.9–51.6)	48.7 (41.8–53.3)	45.4 (39.2–50.7)	0.0002
Male gender	578(86%)	174(83%)	404(87%)	0.238
HIV risk factor				<0.0001
MSM	330(49%)	78(37%)	252(54%)	
Heterosexual	134(20%)	38(18%)	96(21%)	
IDU	88(13%)	49(23%)	39(8%)	
Other/Unknown	123(18%)	44(21%)	79(17%)	
Years since HIV diagnosis	10.3 (5.1–17.3)	13.8 (6.6–22.5)	9.1 (4.8–15.5)	<0.0001
History of AIDS defining events	78(12%)	26(12%)	52(11%)	0.696
Years of ART	6.6 (3.3–14.1)	10.1 (4.0–16.8)	5.7 (3.0–11.9)	<0.0001
NRTI-experience	661(98%)	206(99%)	455(98%)	0.567
NNRTI-experience	326 (48%)	88 (42%)	238 (51%)	0.037
PI-experience	533 (79%)	193 (92%)	340 (73%)	<0.0001
Years with HIV-RNA <50copies/mL	3.07 (1.07–5.62)	2.96 (0.77–6.12)	3.09 (1.25–5.46)	0.493
Time spent with residual viremia (%)	47.8 (23.9–74.1)	51.7 (22.1–74.2)	46.4 (24.3–73.6)	0.437
History of failure to NRTIs	130 (19%)	70 (34%)	60 (13%)	<0.0001
History of failure to NNRTIs	32 (5%)	22 (11%)	10 (2%)	<0.0001
History of failure to PIs	110 (16%)	54 (26%)	56 (12%)	<0.0001
Type of treatment				<0.0001
PI-based	438 (65%)	172 (82%)	266 (57%)	
NNRTI-based	205 (30%)	24 (12%)	181 (39%)	
Other	32 (5%)	13 (6%)	19 (4%)	
Nadir CD4+ count *(cell/μL)*	271 (160–384)	228 (122–336)	289 (205–397)	<0.0001
Zenith HIV-RNA before starting ART *(log*_*10*_ *copies/mL)*	4.85 (4.08–5.32)	4.97 (4.11–5.40)	4.80 (4.08–5.29)	0.187
CD4+ *(cell/μL)*	679 (517–868)	663 (451–854)	687 (542–870)	0.017
HCV-Ab+	159 (24%)	82 (39%)	77 (17%)	<0.0001
ALP *(U/L)*	87 (69–106)	93 (73–110)	86 (69–105)	0.040
ALT *(U/L)*	31 (22–47)	34 (22–63)	30 (22–43)	0.001
Total bilirubin *(mg/dL)*	0.57 (0.34–1.44)	0.68 (0.41–1.71)	0.50 (0.32–1.37)	0.003
FIB-4	0.89 (0.65–1.27)	1.08 (0.72–1.64)	0.84 (0.61–1.12)	<0.0001
eGFR *(ml/min/1.73m* ^*2*^ *)*	104 (93–113)	102 (90–111)	105 (95–114)	0.013
Proteinuria *(mg/dL)*	5 (0–10)	5 (0–10)	5 (0–10)	0.858
Total cholesterol *(mg/dL)*	191 (162–219)	191 (159–216)	191 (164–222)	0.168
LDL cholesterol *(mg/dL)*	119 (95–144)	117 (91–145)	120 (97–144)	0.407
HDL cholesterol *(mg/dL)*	45 (38–55)	42 (36–51)	47 (40–58)	<0.0001
Total/HDL cholesterol	4.25 (3.37–5.18)	4.56 (3.43–5.23)	4.18 (3.36–5.07)	0.045
Triglycerides *(mg/dL)*	122 (86–180)	137 (98–215)	117 (82–166)	0.0002
Glucose *(mg/dL)*	85 (78–93)	87 (80–96)	84 (78–91)	0.001
Hemoglobin *(10* ^*9*^ */L)*	15.1 (14.1–15.7)	14.9 (14.1–15.7)	15.1 (14.2–15.7)	0.315
Phosphate *(mmol/L)*	0.98 (0.86–1.10)	0.99 (0.85–1.13)	0.98 (0.87–10.8)	0.621

*ALP* Alkaline phosphatase, *ALT* Alanine aminotransferase, *ART* Antiretroviral therapy, *eGFR* Estimated glomerular filtration rate, *FIB-4* Liver fibrosis-4 index, *HCV-Ab* Anti-hepatitis C antibodies, *HDL* High density lipoprotein, *IDU* Intravenous drug user, *InSTI* Integrase strand transfer inhibitor, *LDL* Low density lipoprotein, *MSM* Man who have sex with men, *NNRTI* Non-nucleoside reverse transcriptase inhibitor, *NRTIs* Nucleoside reverse transcriptase inhibitors, *PI* Protease inhibitor, *RPV/FTC/TDF* Rilpivirine/tenofovir disoproxil fumarate/emtricitabine, *TDF/FTC* Tenofovir disoproxil fumarate/emtricitabine

After adjusting for age, gender, HIV risk factor, higher HIV-RNA value before starting ART, nadir and baseline CD4+ count, baseline triglycerides and cholesterol, history of failure to nucleoside reverse transcriptase inhibitors (NRTIs) or to non-NRTIs (NNRTIs), opting for an InSTI- rather than a rilpivirine-based regimen was more likely in subjects co-infected with HCV (OR = 2.16; 95%CI = 1.26–3.71; *p* = 0.015), with longer exposure to antiretroviral therapy (OR = 1.05 per year longer; 95%CI = 1.01–1.09; *p* = 0.042) and shorter time with undetectable viremia (OR = 0.93 per year longer; 95%CI = 0.86–0.99; *p* = 0.041), treated with protease inhibitors (PIs) vs NNRTIs (OR = 5.14; 95%CI = 3.09–8.95;*p* = 0.003), and treated with regimens not based on a PI/r and not based on a NNRTI vs those treated with NNRTIs (OR = 5.67; 95%CI = 2.22–14.38;*p* = 0.032).

**Fig. 1:**
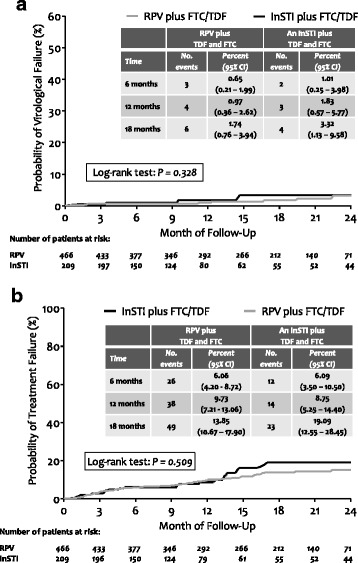
Cumulative probabilities of virological failure (Panel **a**) and of treatment failure (Panel **b**) after switch to a rilpivirine- and or to an integrase inhibitor (InSTI)-based regimen. RPV: rilpivirine; InSTI: integrase strand transfer inhibitor; FTC: emtricitabine; TDF: ftenofovir disoproxil fumarate

The median (interquartile range, IQR) follow-up in the rilpivirine and in the InSTI group was 16.7 (8.8–22.2) and 10.4 (5.4–19.6) months. Four (0.9%) and three (1.4%) patients showed VF at 1 year in the rilpivirine and in the InSTI group; cumulatively, 12 patients (8 [1.7%] in the rilpivirine and 4 [1.9%] in the InSTI group) showed VF during follow-up: their characteristics at VF are detailed in Table 2. TFs at 1 year were 38 (8.2%) and 14 (6.7%) in the rilpivirine and in the InSTI group. The 1-year cumulative probabilities (95%CI) of VF and TF were 0.97% (0.36%–2.62%) and 9.73% (7.21%–13.06%) in the rilpivirine group and 1.83% (0.57%–5.77%) and 8.75% (5.25%–14.4%) in the InSTI group, with no difference between groups (*p* = 0.328 and 0.209 for VF and TF; Fig. 1, panel A and B).

**Table 2:** Patients’ characteristics at virological failure

Patient ID	Previous resistance mutations	Nadir CD4+ (cells/μL)	HIV-RNA zenith (copies/mL)	CD4 + (cells/μL) at baseline	HIV-RNA at baseline	3rd drug	CD4 + (cells/μL) at failure	HIV-RNA (copies/mL) at failure	History of NRTI failure	History of NNRTI failure	History of PI failure	NRTI mutations	NNRTI mutations	PI mutations	InSTI mutations
1412	N/A	258	500.000	675	Undetectable	RPV	691	69	Yes	No	Yes	None	None	None	None
1428	N/A	200	1.400.000	700	Residual viremia	RAL	356	11.236	Yes	Yes	Yes	M41 L, M184 V, L210 W, T215Y	K103 N, V108I	None	N155H
2718	N/A	245	350.000	922	Undetectable	RPV	660	27.823	No	No	No	M41 L, K65R, D67N, M184 V	Y181V	M46I	None
4418	N/A	229	751.000	674	Residual viremia	RPV	578	4.737	No	No	No	A62V, K65R, M184 V	V106I, E138K, H221Y	None	N/A
5443	N/A	113	1.978.100	694	Residual viremia	RPV	648	367	No	No	No	N/A	N/A	N/A	N/A
5799	N/A	324	70.000	653	Residual viremia	RAL	594	138	Yes	No	No	N/A	N/A	N/A	None
5967	N/A	182	78,674	1166	Undetectable	RPV	1083	326	No	No	No	D67N, K70R, M184 V, T215I, K219E	L100I, K103 N, V179 T	None	None
6264	N/A	374	570	904	Residual viremia	RAL	697	216	No	No	No	N/A	N/A	N/A	N/A
7165	None	488	1.492.000	995	Undetectable	RPV	1224	181	No	No	No	N/A	N/A	N/A	N/A
8270	None	108	374.400	444	Undetectable	RPV	598	89	Yes	No	Yes	N/A	N/A	N/A	N/A
9037	None	283	46.291	921	Undetectable	RPV	1067	104	No	No	No	N/A	N/A	N/A	N/A
9827	N/A	95	279	113	Residual viremia	EVG/COBI	174	52	No	No	No	N/A	N/A	N/A	None

*EVG/COBI* Elvitegravir/cobicistat, *InSTI* Integrase strand transfer inhibitor, *N/A* Not available, *NNRTI* Non-nucleoside reverse transcriptase inhibitor, *NRTIs* Nucleoside reverse transcriptase inhibitors, *PI* Protease inhibitor, *RAL* Raltegravir, *RPV* Rilpivirine

The incidence rate (IR) (95%CI) of viral blips was 4.46 (3.07–6.27) and 4.48 (2.51–7.40) per 1000 person months of follow-up in the rilpivirine and in the InSTI group (*p* = 0.988).

The proportion of time spent with residual viremia was comparable in the two groups (9% [IQR 0.5%–49%] and 17% [IQR 0.5%–50%] in the rilpivirine and in the InSTI group, *p* = 0.087).

Overall, 62/466 (13.3%) and 41/209 (19.6%) patients in the rilpivirine and in the InSTI group discontinued at least one drug of the regimen for any reason. Discontinuations were due to toxicity in 34 (7%) and in 17 (8%) patients in the rilpivirine and in the InSTI group. Of the 51 discontinuations occurred because of toxicity, 28 (55%) were deemed tenofovir toxicity (15 [3.2%] and 13 [6.2%] in the rilpivirine and in the InSTI group); other leading causes of discontinuation were non-tenofovir related toxicity or untoward drug interactions (19 [4.1%] and 4 [1.9%] in the rilpivirine and in the InSTI group) and treatment simplification (none and 12 [5.7%] in the rilpivirine and in the InSTI group).

Non-tenofovir related toxicities leading to discontinuation were: liver toxicity in 9 (2%), gastrointestinal toxicity in 5 (1%), central nervous system toxicity in 4 (1%) and undefined toxicity in one further patient in the rilpivirine group, liver toxicity in 1 (<1%) and central nervous system toxicity in 1 (<1%) in the InSTI group. Three (1.5%) patients in the InSTI group discontinued for untoward drug interactions.

Of note, discontinuations occurred in 37/82 (45%) patients who started raltegravir; 27 of these 37 patients (73%) discontinued only raltegravir; the main reason for raltegravir discontinuation was treatment simplification (12/27 [44%]).

By the multivariable Cox regression model, TF was independently associated with being on therapy with a PI vs. a NNRTI at switch (AHR = 0.52; 95%CI = 0.31–0.90; *p* = 0.018), baseline total/HDL-cholesterol ratio (AHR = 1.19 per 0.5-units increments; 95%CI = 1.06–1.34; *p* = 0.004), baseline eGFR (AHR = 0.78 per 10-units increments; 95%CI = 0.67–0.90; *p* = 0.001) and baseline hemoglobin (AHR = 0.78 per 1-unit increments; 95%CI = 0.64–0.94; *p* = 0.009), whereas treatment group (rilpivirine vs. InSTI), HCV-coinfection, nadir and baseline CD4+ cell count, time with HIV-RNA <50 copies/mL, time spent with residual viremia, years of antiretroviral therapy, failure to NRTIs, failure to NNRTIs, failure to PIs, triglycerides and FIB-4 were not. No violation of the proportional hazard assumption was detected using graphical representation (Log-log plot).

## Discussion

In this non-randomized study, the efficacy of switching to a FDC of rilpivirine/tenofovir disoproxil fumarate/emtricitabine was similar to that of switching to an InSTI plus tenofovir disoproxil fumarate/emtricitabine-based regimen: both the virological outcomes and treatment discontinuations were similar. As regards to virological outcomes, not only the cumulative risk of virological failure was very low and similar for both regimens, but also the incidence of viral blips and the exposure to residual viremia during follow-up were not statistically different. This suggest that both of these types of switch regimen can be safely used in clinical practice.

However, among patients switched to InSTI, most discontinuation occurred in those switched to raltegravir: this suggests clinician should preferentially switch patients virologically suppressed to a once-daily regimen.

A lower risk of failure was observed in patients switched from PIs: our hypothesis is that patients switched from a PI to a PI-sparing regimen have a greater improvement in symptoms than those switched from NNRTI to another NNRTI or to a InSTI-based regimen. Indeed, the only switch study in which the superiority of the switch strategy was demonstrated was the STRATEGY-PI [6]: in this randomized clinical trial, patients who underwent treatment switch to a FDC of elvitegravir/cobicistat/tenofovir disoproxil fumarate/emtricitabine had a significant improvement in several PI-related symptoms (mainly gastrointestinal). On the contrary, in studies with comparable design, switching from a NNRTI to a FDC of elvitegravir/cobicistat/tenofovir disoproxil fumarate/emtricitabine was not superior to continuing the NNRTI [4], as well as switching from a FDC of efavirenz/tenofovir disoproxil fumarate/emtricitabine to rilpivirine/tenofovir alafenamide/emtricitabine did not resulted superior to continuing efavirenz/tenofovir disoproxil fumarate/emtricitabine [2].

Although nonspecific, hemoglobin is a marker of the general health status; it has been also associated with mortality in HIV-infected patients [10]: thus, it not surprising that in our analysis it was independently associated with TF, as it is conceivable that patients with a worse general health status are more prone to interrupt or change drugs for toxicity issues. Patients with a higher baseline total/HDL-cholesterol ratio are those with greater metabolic problems: it is likely that, in these cases, treatment was then further modified, in the attempt of normalizing the dyslipidemia. The greater risk of TF in patients with lower eGFR value is consistent with the observation that a major cause of TF in our study was toxicity due to TDF.

Overall, the results of our study confirm, in a large, unselected population, the safety and the efficacy of switch regimens based on either rilpivirine or InSTI demonstrated in randomized clinical trials [1–6], thus providing useful data for clinical decision making.

With this study, we also aimed at identifying clinical reasons for opting for an InSTI- rather than a rilpivirine-based switch strategy in everyday clinical practice; this because there are no data from randomized clinical trials guiding clinicians in this decision and the results from switch trials do not clear indicate which is the best switching strategy. The results of our analysis showed that we preferentially opted for InSTI in patients with HCV co-infection, a longer ART duration, a shorter time of HIV suppression, an ongoing treatment with PIs. Possible drivers of these preferences are likely related to the common belief that NNRTIs entail greater liver toxicity (at least among persons co-infected with HCV) [11–13], to the results of the STRATEGY-PI study (which showed the superiority of this strategy compared to continuing on a PI) [6] and to the belief that InSTI are more potent than NNRTIs, thus favoring this strategy in patients a shorter time of viral suppression when a decision on whether or not switching has to be taken.

Major limitations of this study are the lack of randomization and the fact that the patients switched to rilpivirine/tenofovir disoproxil fumarate/emtricitabine were different from those switched to an InSTI in many baseline clinical features; however, after adjustment for these differences, the multivariable analysis confirmed that switching to a regimen rather than the other was not independently associated to TF.

## Conclusions

In our clinical practice, the durability of switch regimens based on rilpivirine and on InSTI (along with TDF and FTC) was comparable and both showed a very low probability of VF.

## Additional file

Additional file 1: Specific PIs and NNRTIs ongoing at baseline. (DOCX 12 kb)

## Abbreviations

AHR: Adjuster hazard ratio
ALP: Alkaline phosphatase
ALT: Alanine aminotransferase
ART: Antiretroviral therapy
AST: Aspartate aminotransferase
CI: Confidence interval
CKD-EPI: Chronic Kidney Disease Epidemiology Collaboration Equation
eGFR: Estimated glomerular filtration rate
EVG/COBI: Elvitegravir/cobicistat
FDC: Fixed dose combination
FIB-4: Liver fibrosis-4 index
HCV-Ab: Anti-hepatitis C antibodies
HDL: High density lipoprotein
IDD-HSR: Infectious Diseases Department San Raffaele Hospital
IDU: Intravenous drug user
InSTI: Integrase strand transfer inhibitor
IQR: Interquartile range
IR: Incidence rate
LDL: Low density lipoprotein
MSM: Man who have sex with men
N/A: Not available
NNRTI: Non-nucleoside reverse transcriptase inhibitor
NRTIs: Nucleoside reverse transcriptase inhibitors
OR: Odds ratio
PCR: Polymerase chain reaction
PI: Protease inhibitor
PI/r: Ritonavir-boosted protease inhibitor
RAL: Raltegravir
RPV: Rilpivirine
RPV/FTC/TDF: Rilpivirine/tenofovir disoproxil fumarate/emtricitabine
TDF/FTC: Tenofovir disoproxil fumarate/emtricitabine
TF: Treatment failure
VF: Virological failure

## References

[1] Palella FJ, Fisher M, Tebas P, Gazzard B, Ruane P, Van Lunzen J (2014). Simplification to rilpivirine/emtricitabine/tenofovir disoproxil fumarate from ritonavir-boosted protease inhibitor antiretroviral therapy in a randomized trial of HIV-1 RNA-suppressed participants. AIDS.

[2] DeJesus E, Ramgopal M, Crofoot G, Ruane P, LaMarca A, Mills A, Martorell CT, de Wet J, Stellbrink HJ, Molina JM, Post FA, Valero IP, Porter D, Liu Y, Cheng A, Quirk E, SenGupta D, Cao H (2017). Switching from efavirenz, emtricitabine, and tenofovir disoproxil fumarate to tenofovir alafenamide coformulated with rilpivirine and emtricitabine in virally suppressed adults with HIV-1 infection: a randomised, double-blind, multicentre, phase 3b, non-inferiority study. Lancet HIV.

[3] Martínez E, Larrousse M, Llibre JM, Gutiérrez F, Saumoy M, Antela A (2010). Substitution of raltegravir for ritonavir-boosted protease inhibitors in HIV-infected patients: the SPIRAL study. AIDS.

[4] Pozniak A, Markowitz M, Mills A, Stellbrink HJ, Antela A, Domingo P (2014). Switching to coformulated elvitegravir, cobicistat, emtricitabine, and tenofovir versus continuation of non-nucleoside reverse transcriptase inhibitor with emtricitabine and tenofovir in virologically suppressed adults with HIV (STRATEGY-NNRTI): 48 week results of a randomised, open-label, phase 3b non-inferiority trial. Lancet Infect Dis.

[5] Trottier B, Lake JE, Logue K, Brinson C, Santiago L, Brennan C, Koteff JA, Wynne B, Hopking J, Granier C, Aboud M. Dolutegravir/abacavir/lamivudine versus current ART in virally suppressed patients (STRIIVING): a 48-week, randomized, non-inferiority, open-label, phase IIIb study. Antivir Ther. 2017. doi:10.3851/IMP3166 [Epub ahead of print]. 10.3851/IMP3166 28401876

[6] Arribas JR, Pialoux G, Gathe J, Di Perri G, Reynes J, Tebas P (2014). Simplification to coformulated elvitegravir, cobicistat, emtricitabine, and tenofovir versus continuation of ritonavir-boosted protease inhibitor with emtricitabine and tenofovir in adults with virologically suppressed HIV (STRATEGY-PI): 48 week results of a randomised, open-label, phase 3b, non-inferiority trial. Lancet Infect Dis.

[7] Eron JJ, Young B, Cooper DA, Youle M, Dejesus E, Andrade-Villanueva J (2010). Switch to a raltegravir-based regimen versus continuation of a lopinavir-ritonavir-based regimen in stable HIV-infected patients with suppressed viraemia (SWITCHMRK 1 and 2): two multicentre, double-blind, randomised controlled trials. Lancet.

[8] Levey AS, Stevens LA, Schmid CH, Zhang YL, Castro AF, Feldman HI (2009). A new equation to estimate glomerular filtration rate. Ann Intern Med.

[9] Sterling RK, Lissen E, Clumeck N, Sola R, Correa MC, Montaner J (2006). Development of a simple noninvasive index to predict significant fibrosis in patients with HIV/HCV coinfection. Hepatology.

[10] Tate JP, Justice AC, Hughes MD, Bonnet F, Reiss P, Mocroft A (2013). An internationally generalizable risk index for mortality after one year of antiretroviral therapy. AIDS.

[11] Servoss JC, Kitch DW, Andersen JW, Reisler RB, Chung RT, Robbins GK (2006). Predictors of antiretroviral-related hepatotoxicity in the adult AIDS clinical trial group (1989-1999). J Acquir Immune Defic Syndr.

[12] Brück S, Witte S, Brust J, Schuster D, Mosthaf F, Procaccianti M (2008). Hepatotoxicity in patients prescribed efavirenz or nevirapine. Eur J Med Res.

[13] Soriano V, Puoti M, Garcia-Gascó P, Rockstroh JK, Benhamou Y, Barreiro P (2008). Antiretroviral drugs and liver injury. AIDS.

