# Supplementary material for: Durability of switch regimens based on rilpivirine or on integrase inhibitors, both in association with tenofovir and emtricitabine, in HIV-infected, virologically suppressed patients
Source: BMC Infect Dis. 2017 Nov 16;17:723. doi: 10.1186/s12879-017-2831-9 (PMC5691866; doi:10.1186/s12879-017-2831-9)
Supplement: Additional file 1: — Specific PIs and NNRTIs ongoing at baseline. (DOCX 12 kb) [file 12879_2017_2831_MOESM1_ESM.docx]

Supplementary material. Specific PIs and NNRTIs ongoing at baseline.

|  | InSTI + TDF/FTC  (n=209) | RPV/FTC/TDF  (n=466) |
| --- | --- | --- |
| Atazanavir | 66 (32%) | 139 (30%) |
| Darunavir | 54 (26%) | 76 (16%) |
| Fosamprenavir | 18 (9%) | 4 (1%) |
| Lopinavir | 33 (16%) | 0 |
| Saquinavir | 1 (0.5%) | 0 |
| Efavirenz | 19 (9%) | 155 (33%) |
| Nevirapine | 4 (2%) | 20 (4%) |
| Etravirine | 0 | 4 (1%) |
| Rilpivirine | 1 (0.5%) | 0 |
| Lersivirine | 0 | 2 (0.5%) |
